# RISK AND PROTECTIVE FACTORS OF COGNITIVE FUNCTIONING AND DEMENTIA: COMPARING MEXICAN AND NON-MEXICAN HISPANICS

**DOI:** 10.1093/geroni/igad104.3576

**Published:** 2023-12-21

**Authors:** Nasim Ferdows

**Affiliations:** Northeastern University, Boston, Massachusetts, United States

## Abstract

This study investigates the cognitive functioning of Hispanic individuals aged 65 and above, focusing on the unique risk and protective factors associated with dementia in Mexican Hispanics and non-Mexican Hispanics. The study utilizes data from the Harmonized Cognitive Assessment Protocol (HCAP) subsample of the Health and Retirement Study (HRS), comprising 388 Hispanic older individuals who participated in the HCAP in 2016 (traced back to 2000), allowing for a comprehensive examination of cognitive health within this population. Cognitive function was measured using the Mini-Mental State Examination (MMSE) score and to classify dementia. The findings reveal distinct patterns in cognitive functioning between Mexican Hispanic and non-Mexican Hispanic populations. Mexican Hispanic males exhibit lower MMSE scores across all age groups, while Mexican Hispanic females show slightly higher scores compared to non-Mexican Hispanic females. Education and wealth levels do not significantly affect cognitive scores in either group. Mexican Hispanics have lower odds of dementia compared to non-Mexican Hispanics, even when accounting for sociodemographic, midlife, and late-life characteristics. Both educational attainment and wealth emerge as protective factors against dementia for both groups. Additionally, midlife obesity, hearing loss, and later-life physical inactivity are identified as specific risk factors for dementia in Mexican Hispanics but have an insignificant impact on non-Mexican Hispanics. These findings have implications for targeted interventions and policies to promote cognitive well-being and address health disparities in older Hispanics. The aim is to improve the overall quality of life and achieve equitable healthcare outcomes.

